# Contrasting outcomes of visceral leishmaniasis in two patients with membranous nephropathy receiving immunosuppressive therapy: a case report

**DOI:** 10.3389/fimmu.2026.1889409

**Published:** 2026-08-12

**Authors:** Xiu Sun, Yan Wang, Ruixian Duan

**Affiliations:** Department of Infection, Shanxi Bethune Hospital, Shanxi Academy of Medical Sciences, Third Hospital of Shanxi Medical University, Tongji Shanxi Hospital, Taiyuan, China

**Keywords:** case report, hemophagocytic lymphohistiocytosis, immunosuppressive therapy, leflunomide, membranous nephropathy, visceral leishmaniasis

## Abstract

**Background:**

Visceral leishmaniasis (VL) often presents with an atypical and fulminant course in immunocompromised hosts, and treatment faces multiple challenges including drug toxicity, immunosuppressive status, and complications.

**Case presentation:**

Here we report two cases of VL in patients with membranous nephropathy(MN) who developed the infection after long-term immunosuppressive therapy. Both patients presented with fever, pancytopenia, and markedly elevated inflammatory markers. Case 1 had relapsed VL complicated by pre-existing renal insufficiency, experienced disease relapse after anti-parasitic treatment, and ultimately died of multi-organ failure. Case 2 developed hemophagocytic lymphohistiocytosis (HLH) but achieved clinical reversal after the addition of glucocorticoids to anti-parasitic therapy. Both patients received standard treatment with amphotericin B cholesteryl sulfate complex (ABCD). However, Case 1 with pre-existing renal insufficiency eventually died, whereas Case 2 with a milder degree of immunosuppression was successfully cured.

**Conclusion:**

These cases suggest that when patients with MN develop VL after long-term immunosuppressive therapy, treatment faces multiple challenges including renal insufficiency, depth of immunosuppression, and secondary HLH, necessitating individualized and comprehensive management.

## Introduction

Visceral leishmaniasis (VL) is a vector-borne parasitic disease caused by *Leishmania* protozoa and transmitted by sandflies, which is endemic in many regions worldwide (1–3).Its classic clinical manifestations include prolonged irregular fever, hepatosplenomegaly, and pancytopenia, but the clinical presentation is heterogeneous, ranging from asymptomatic infection to life-threatening severe disease (2). In non-endemic areas, VL is frequently overlooked due to low disease prevalence and limited clinical suspicion (4), often leading to diagnostic delays and misdiagnosis, particularly in immunocompromised individuals (5). Immunosuppressive therapy, such as long-term use of glucocorticoids, conventional immunosuppressive agents, or biological agents, for the management of autoimmune diseases or solid organ transplantation, is a well-established risk factor for the occurrence, relapse, or complicated clinical course of VL (3, 6). It not only increases host susceptibility but also impairs parasite clearance and may trigger life-threatening complications such as hemophagocytic lymphohistiocytosis (HLH) (7). HLH is a life-threatening hyperinflammatory syndrome characterized by uncontrolled activation of T lymphocytes, natural killer cells, and macrophages in response to continuous antigen stimulation, resulting in a cytokine storm and immune-mediated injury of multiple organ systems (8, 9). This dysregulated activation arises from impaired cytotoxic function of NK cells and CD8+ T lymphocytes, which prevents effective elimination of the triggering antigen and sustains the hyperinflammatory response (10, 11). Its diagnosis is based on the HLH-2004 criteria, which require at least five of eight features: fever, splenomegaly, cytopenias (affecting at least two lineages), hypertriglyceridemia or hypofibrinogenemia, hemophagocytosis in bone marrow or other tissues, low or absent NK cell activity, hyperferritinemia, and elevated soluble CD25 (12). Extreme hyperferritinemia (>10,000 ng/mL) is a particularly important diagnostic clue for HLH in adults, with a reported sensitivity of 87%-91% (13). In immunocompromised patients, VL can present with severe manifestations, including atypical forms, disseminated infections, and poor treatment response, reflecting impaired control of intracellular parasite replication (3). This may lead to prolonged antigenic exposure, which—given that VL is a recognized trigger of secondary HLH (14, 15) —may facilitate the development of HLH in such immunocompromised patients. VL-associated HLH (VL-HLH) is a severe complication of VL that is particularly challenging to recognize and manage, as the clinical and laboratory features of VL and HLH overlap substantially. Beyond fever, cytopenias, and hepatosplenomegaly, both conditions may present with marked hyperferritinemia and, in some cases, hemophagocytosis on bone marrow examination. Given these overlapping features, patients with severe VL may fulfill the HLH-2004 criteria, placing them at risk of misdiagnosis and inappropriate immunosuppressive therapy (16). Immunochemotherapy directed exclusively at HLH may be ineffective or even detrimental, whereas prompt anti-parasitic therapy must be prioritized (17, 18).

Membranous nephropathy (MN), one of the leading causes of nephrotic syndrome in adults and accounting for approximately 20% to 30% of primary glomerulonephritis cases, is typically managed with long-term glucocorticoids and immunosuppressive agents (e.g., cyclophosphamide, tacrolimus, leflunomide) (19), making these patients vulnerable to opportunistic infections. VL can affect the kidneys, with reported manifestations including acute kidney injury, proteinuria, hematuria, and histopathological patterns such as glomerulonephritis and interstitial nephritis (20). However, while the existing literature has largely focused on VL-associated renal impairment, reports of VL occurring secondary to immunosuppressive therapy in MN patients remain exceedingly rare.

Herein, we present two cases of VL complicating immunosuppressive therapy in patients with MN. Both individuals had a history of prolonged immunosuppression but exhibited contrasting clinical outcomes: one experienced disease relapse following anti-parasitic treatment and ultimately succumbed to multi-organ failure, whereas the other was diagnosed with HLH in a timely manner during anti-parasitic therapy and, after receiving adjunctive immunotherapy, achieved full recovery. Despite the growing recognition of VL as an opportunistic infection in immunocompromised populations, data on its clinical course and optimal management in patients with MN—a condition requiring long-term immunosuppressive therapy—remain scarce. In particular, little is known about the factors that determine divergent outcomes, the role of HLH as a complicating factor, and the optimal approach to balancing anti-parasitic therapy with immunomodulation in this specific population. By comparing the clinical characteristics, diagnostic and therapeutic processes, and outcomes of these two patients, this article aims to (1): characterize the clinical course of VL in MN patients on immunosuppressive therapy, with particular emphasis on the challenges posed by pre-existing renal insufficiency during anti-parasitic treatment (2); highlight the challenges of managing VL-HLH in immunosuppressed patients; and (3) provide practical insights for early recognition and individualized management of VL in immunosuppressed patients, particularly in those with renal impairment in non-endemic regions.

## Case presentation

### Case one

**Figure 1:**
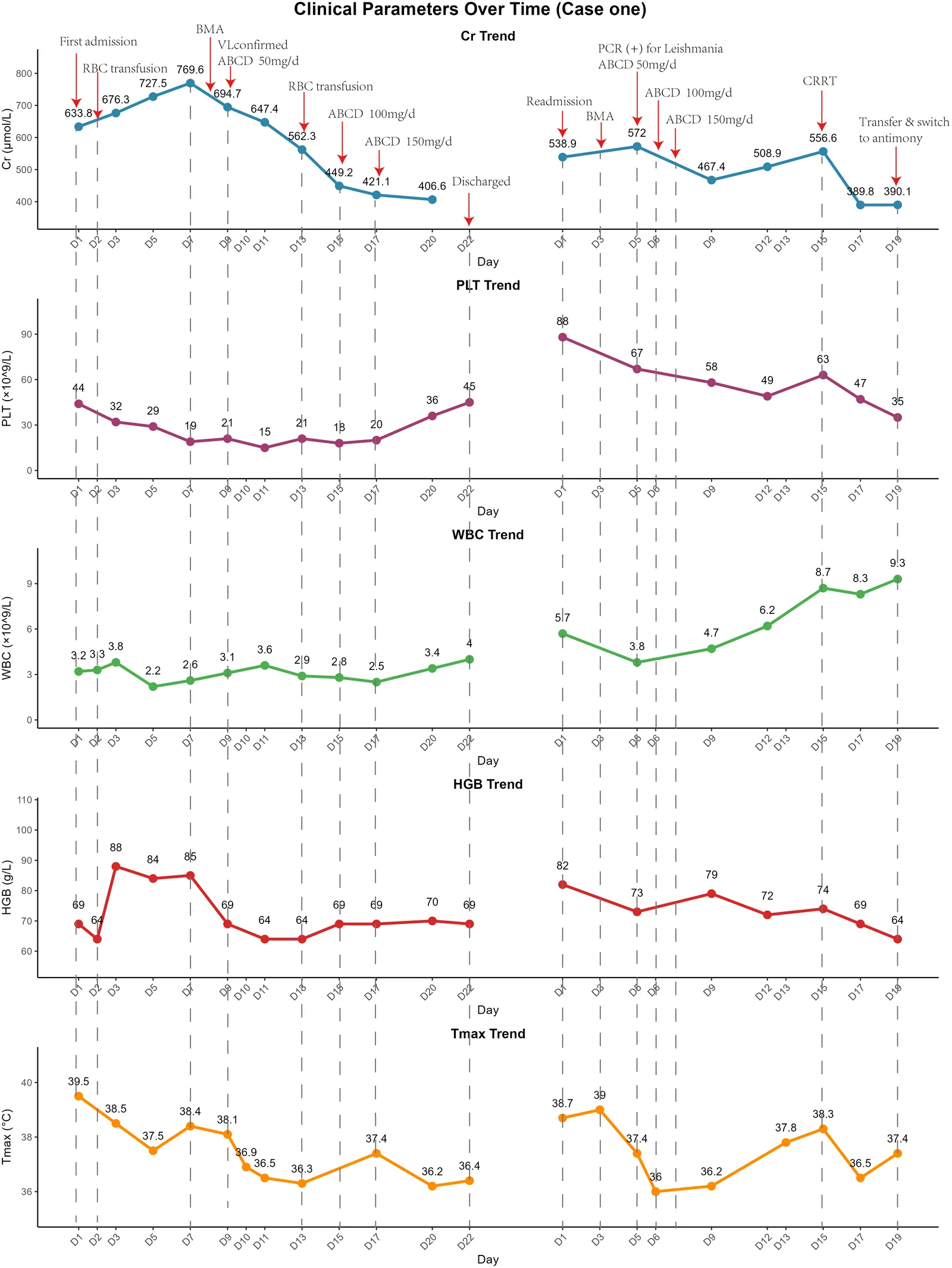
Clinical course of Case 1 from admission to transfer. From top to bottom, the five panels display serum creatinine (μmol/L), platelet count (×10^9^/L), white blood cell count (×10^9^/L), hemoglobin (g/L) over time (days), and daily peak body temperature (°C). Gray vertical dashed lines and red arrows indicate key clinical events. Initial empirical antibiotic therapy was ineffective; creatinine peaked at 769.6 μmol/L and platelets dropped to a nadir of 19×10^9^/L. On day 9 of admission, BMA confirmed the diagnosis of VL. ABCD was initiated at 50 mg/day (days 9–14), increased to 100 mg/day (days 15–16), and then escalated to 150 mg/day (days 17–22). Creatinine declined to 406.6 μmol/L, platelets recovered to 45×10^9^/L, and fever resolved, allowing discharge on day 22. Packed red blood cell transfusions (2 units each) were administered on days 2 and 13 of the first admission. However, the patient was readmitted on day 5 post-discharge with recurrent fever; BMA on day 3 of readmission confirmed persistent VL. ABCD was resumed at 50 mg/day (day 5 of readmission), 100 mg/day (day 6), and 150 mg/day (from day 7 onward). Renal function initially improved but subsequently deteriorated, with creatinine peaking at 556.6 μmol/L and urine output dropping to approximately 300 mL/day, prompting initiation of CRRT on day 15 of readmission. Despite this, the patient developed severe metabolic acidosis and refractory circulatory failure, and was transferred to a higher-level facility on day 19 of readmission. ABCD, amphotericin B cholesteryl sulfate complex; BMA, bone marrow aspiration; VL, visceral leishmaniasis; CRRT, continuous renal replacement therapy.

A 57-year-old male patient (weighing 60.6 kg) with a 20-year history of MN, who had been receiving immunosuppressive therapy (most recently prednisone acetate 15 mg/d combined with leflunomide 20 mg/d prior to admission, presented with a baseline serum creatinine level of approximately 300 μmol/L. He was admitted with a chief complaint of bilateral lower extremity weakness of one week’s duration. Upon admission, his vital signs were stable, and physical examination was unremarkable in the cardiopulmonary, abdominal, and lower extremity systems. Abdominal ultrasound revealed no hepatosplenomegaly. Laboratory investigations showed pancytopenia (white blood cell count 3.2×10^9^/L, red blood cell count 2.55×10¹²/L, hemoglobin 69 g/L, platelet count 44×10^9^/L), elevated inflammatory markers (C-reactive protein 73.01 mg/L, ferritin 2194 ng/mL), renal impairment (urea 33.6 mmol/L, creatinine 633.8 μmol/L, urine protein 3+), coagulation abnormalities (prothrombin time 9.2 s, fibrinogen 2.96 g/L, D-dimer 11695 ng/mL), and liver dysfunction (alanine aminotransferase 57.1 IU/L, aspartate aminotransferase 63.5 IU/L, albumin 24.8 g/L, total bilirubin 9.8 μmol/L, lactate dehydrogenase 901 IU/L), and hypertriglyceridemia (triglycerides 4.55 mmol/L). Reference ranges for all parameters are provided in **Table 1**. Upon admission, the patient received a transfusion of 2 units of packed red blood cells to correct anemia. During hospitalization, the patient developed a fever with a maximum temperature of 39.5°C, accompanied by melena, and all immunosuppressive agents were discontinued. Follow-up laboratory tests performed on day 7 of admission (4 days after blood transfusion) revealed disease progression: white blood cell count decreased to 2.6×10^9^/L, hemoglobin 85 g/L, platelet count to 19×10^9^/L, C-reactive protein 114.64 mg/L, serum creatinine 769.6μmol/L, urea 25.0 mmol/L. On day 13 of the first admission, he received a second transfusion of packed red blood cells, but hemoglobin improvement was not significant (**Figure 1**). To elucidate the cause of fever and hematologic abnormalities, a bone marrow aspiration (BMA) was performed, which revealed *Leishmania* amastigotes on smear, leading to a confirmed diagnosis of VL. The patient resided in Xiyang County, Jinzhong City, Shanxi Province—a non-endemic county for VL, but geographically adjacent to Yangquan City, an area with documented VL transmission—and had no recent travel history to other endemic regions. Immunological assessment performed at the time of diagnosis revealed a CD4+ T-cell count of 247 cells/μL, with corresponding cytokine levels of IL-6 5.11 pg/mL, IL-8 37.15 pg/mL, IL-10 12.88 pg/mL, and IFN-γ 2.86 pg/mL. The patient met four of the eight HLH-2004 diagnostic criteria (fever, cytopenias involving two lineages, hypertriglyceridemia, and hyperferritinemia), but did not reach the diagnostic threshold for secondary HLH. Splenomegaly was not detected, hemophagocytosis was not observed on bone marrow examination, and NK cell activity and sIL-2R were not measured. He was initiated on anti-parasitic therapy with amphotericin B cholesteryl sulfate complex (ABCD, a lipid formulation of amphotericin B) at an initial dose of 50 mg/day (approximately 0.8 mg/kg/day). On the second day of treatment, body temperature normalized. After six days of treatment, serum creatinine decreased to 449.2μmol/L. The dose was then escalated to 100 mg/day(1.7 mg/kg/day) for two days, with a further reduction in creatinine to 421.1μmol/L. Subsequently, the dose was increased to 150 mg/day (2.5 mg/kg/day) for six days, after which the patient was discharged in stable condition with normal vital signs; his serum creatinine level of 406.6μmol/L. The cumulative ABCD dose during the first admission was 1,400 mg (23.1 mg/kg). The clinical course during the first admission, including peak body temperature, serum creatinine levels, platelet count, and the ABCD dosing schedule, is shown in the left portion of **Figure 1**.

**Table 1:** Summary of clinical characteristics, treatment, and outcomes of Case 1 and Case 2.

Feature	Case 1	Case 2	Reference range
Demographics			
Age/Sex	57 years / Male	69 years / Male	-
Underlying disease	Membranous nephropathy	Membranous nephropathy	-
Prior immunosuppressive regimen	Prednisone acetate 15 mg/d+ Leflunomide 20 mg/day	Prednisone acetate 25 mg/day + Tacrolimus 2.5mg/day	-
Immunosuppressant status	Continued until admission	Self-discontinued 20 days before admission	-
Baseline laboratory findings			
WBC (×10^9^/L)	3.2	2.5	3.5-9.5
RBC (×10^12^/L)	2.55	3.7	4.30-5.80
HGB (g/L)	69	101	130-175
PLT (×10^12^/L)	44	20	125-350
CRP ( mg/L)	73.01	44.4	0.0-8.0
ALT (IU/L)	57.1	94.9	Sep-50
AST (IU/L)	63.5	209.4	15-40
ALB(g/L)	24.8	21.8	40-55
TBil(μmol/L)	9.8	14.3	≤26
TG(mmol/L)	4.55	1.88	≤1.7
LDH(IU/L)	901.7	678.4	120-250
urea(mmol/L)	33.6	7.3	3.1-8.0
Cr (μmol/L)	633.8	90.6	72-127
Ferritin (ng/mL)	2,194	11,197	23.9-336.2
PT (s)	9.2	14.3	9.4-12.5
FIB(g/L)	2.96	1.25	2.38-4.98
D-dimer (ng/mL)	11695	10197	0-243
IL-6(pg/mL)	5.11	104.06	0-5.3
IL-8(pg/mL)	37.15	103.05	0-20.6
IL-10(pg/mL)	12.88	160.94	0-4.91
IFN-γ(pg/mL)	2.86	24.65	0-7.42
CD4+ T-cell count(cells/μL)	247	-	340-1072
sIL-2R(pg/mL)	-	7355.2	25.65-313.37
CD3+ T lymphocytes (%)	-	92.81	50.62-85.22
CD3+CD8+ perforin expression (%)	-	88.4	27.98-75.18
CD3+CD8+granzyme B expression (%)	-	91.92	26.33-79.73
NK cells(%)	-	1.01	3.06-36.21
**HLH**	No (4/8)	Yes	-
Treatment			
Anti-parasitic therapy	ABCD, 50→100→150 mg/d	ABCD, 50→100→150 mg/d	-
Immunomodulatory therapy	None	Methylprednisolone 40 mg/day × 7 days	-
Renal replacement therapy	CRRT	None	-
Outcome			
Initial response	Effective (fever resolved, creatinine declined)	Ineffective (persistent fever)	-
Final outcome	Initial improvement → subsequent deterioration → transferred → deceased	Discharged in stable condition	-

WBC, white blood cell count; RBC, red blood cell count; HGB, hemoglobin; PLT, platelet count; CRP, C-reactive protein; ALT, alanine aminotransferase; AST, aspartate aminotransferase; ALB, albumin; TBil, total bilirubin; TG, triglycerides; LDH, lactate dehydrogenase; Cr, creatinine; PT, prothrombin time; FIB, fibrinogen; IL, interleukin; IFN-γ, interferon-gamma; sIL-2R, soluble interleukin-2 receptor; HLH, hemophagocytic lymphohistiocytosis; ABCD, amphotericin B cholesterylsulfate complex; CRRT, continuous renal replacement therapy;NK: natural killer.

Five days after discharge, the patient developed fever and diarrhea again and was readmitted. Repeat bone marrow smear showed myeloid hyperplasia, and PCR testing for *Leishmania* DNA in the bone marrow remained positive. He was retreated with ABCD complex at escalating doses: 50 mg/day for one day, followed by 100 mg/day for one day, and then 150 mg/day for 13 days. His body temperature initially normalized but spiked again on the seventh day of treatment. Laboratory findings revealed progressively decreasing albumin levels, reaching a nadir of 11.7 g/L. Renal function initially improved but subsequently deteriorated, with serum creatinine rising to 556.6 μmol/L, and urine output progressively decreased to 300 mL/day. The patient developed severe metabolic acidosis, with arterial blood gas analysis showing pH 7.267, PaCO_2_ 20.9 mmHg, bicarbonate 9.5 mmol/L, serum potassium 2.9 mmol/L, and lactate 0.7 mmol/L. Continuous renal replacement therapy (CRRT) was initiated, but his circulatory status continued to deteriorate. The patient was then transferred to a higher-level referral center, where anti-parasitic therapy was switched to pentavalent antimony based on the decision of the treating physicians at that facility. However, due to his end-stage renal disease and irreversible multiorgan failure, he failed to respond to salvage treatment and died shortly after transfer. The cumulative ABCD dose during the second admission was 2,100 mg (34.7 mg/kg). The changes in key clinical parameters (body temperature, serum creatinine, and platelet count) during the second admission are shown in the right portion of **Figure 1**.

### Case two

**Figure 2:**
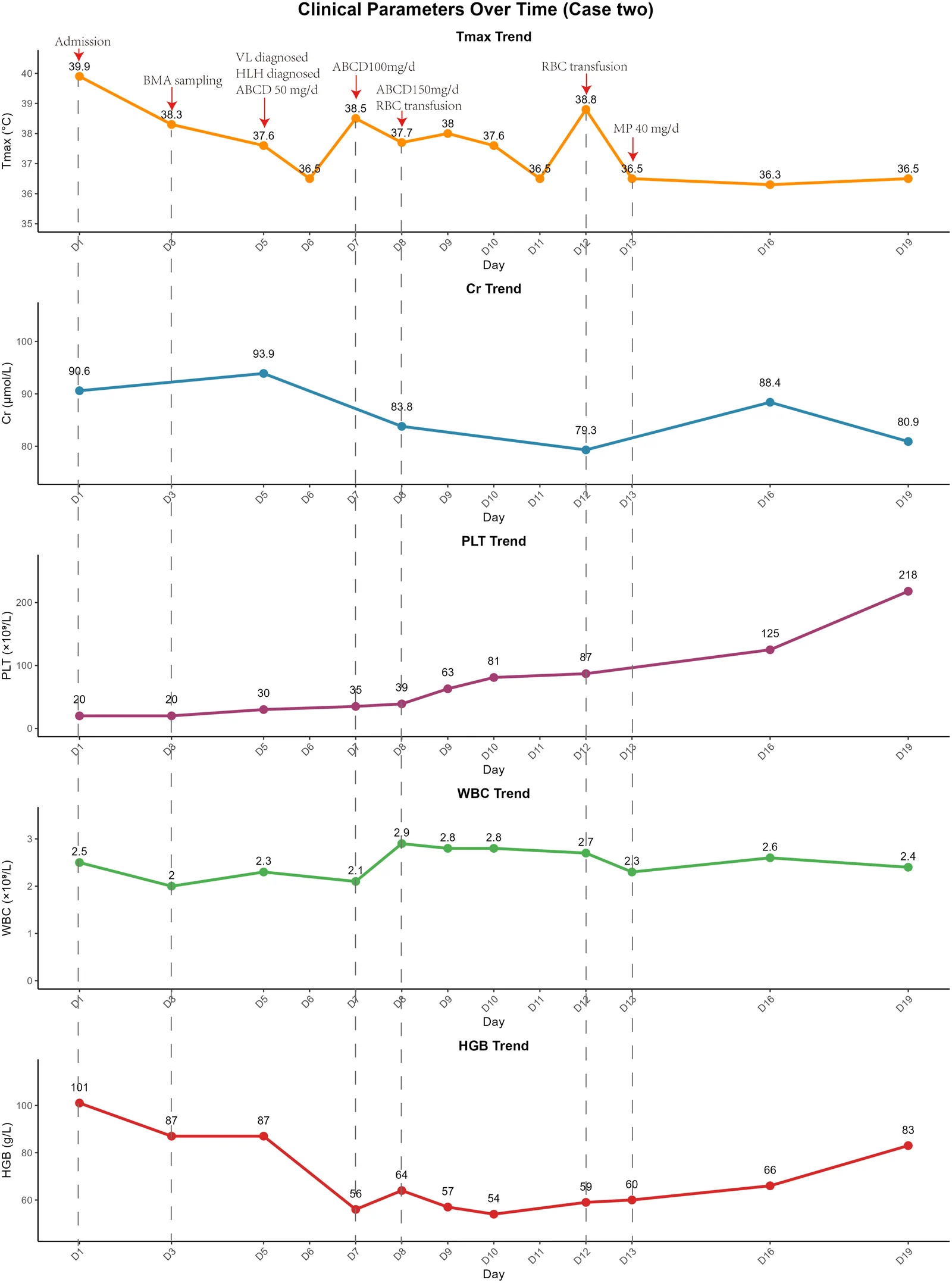
Clinical course of Case 2 during hospitalization. From top to bottom, the five panels display daily peak body temperature (°C), serum creatinine (μmol/L), platelet count (×10^9^/L), white blood cell count (×10^9^/L), and hemoglobin (g/L) over time (days). Gray vertical dashed lines and red arrows indicate key clinical events. The patient presented with fever (up to 39.9°C) and severe thrombocytopenia (platelets 20×10^9^/L). BMA was performed on day 3; on day 5, both serum anti-*Leishmania* antibody test was positive and *Leishmania* amastigotes were identified on bone marrow smear, confirming VL. On the same day, the patient was also diagnosed with HLH (fulfilling HLH-2004 criteria). ABCD was initiated on day 5 at 50 mg/day (days 5-6), increased to 100 mg/day (day 7), and then escalated to 150 mg/day (from day 8 onward). Hemoglobin dropped to 56 g/L on day 7; Packed red blood cell transfusions (2 units each) were administered on days 8 and 12. Despite ABCD therapy, the patient remained febrile. MP was added on day 13. Following MP initiation, fever subsided and the patient was discharged in stable condition. ABCD, amphotericin B cholesteryl sulfate complex; BMA, bone marrow aspiration; VL, visceral leishmaniasis; HLH, hemophagocytic lymphohistiocytosis; MP, methylprednisolone sodium succinate.

A 69-year-old male patient (weighing 75 kg) with a 2.5-year history of MN had previously been treated with oral tacrolimus (2.5 mg/d) combined with prednisone acetate (25 mg/day). Twenty days prior to admission, he discontinued all immunosuppressive agents on his own initiative. He was admitted with a 15-day history of fever, with a maximum temperature of 39.9 °C. On admission, his vital signs were stable. Cardiopulmonary examination revealed no significant abnormalities, while abdominal examination revealed splenomegaly. Laboratory investigations showed pancytopenia (white blood cell count 2.5×10^9^/L, hemoglobin 101 g/L, platelet count 20×10^9^/L), markedly elevated inflammatory markers (C-reactive protein 44.4 mg/L, ferritin 11,197 ng/mL), liver dysfunction (alanine aminotransferase 94.9 IU/L, aspartate aminotransferase 209.4 IU/L, albumin 21.8 g/L,total bilirubin 14.3 μmol/L), lactate dehydrogenase 678.4 IU/L, and triglycerides 1.88 mmol/L. Coagulation abnormalities included a prothrombin time of 14.3 seconds, fibrinogen 1.25 g/L, and markedly elevated D-dimer (10,197 ng/mL). Abdominal ultrasound revealed hepatomegaly, splenomegaly, and an accessory spleen. During hospitalization, the patient developed hypotension and atrial fibrillation. Amiodarone was administered for rhythm conversion, and norepinephrine was used to maintain blood pressure. Serological testing for anti-*Leishmania* antibodies was positive, and BMA smear revealed *Leishmania* amastigotes (Leishman-Donovan bodies), confirming the diagnosis of VL. The patient, a long-term resident of Yangquan City, Shanxi Province—an area with documented VL cases in recent years—had no recent travel history to other endemic regions. Further investigations revealed an elevated soluble interleukin-2 receptor (sIL-2R) level of 7,355.2 pg/mL, CD3+ T lymphocytes accounting for 92.81% of total lymphocytes, natural killer (NK) cells comprising 1.01% of total lymphocytes, and CD3+CD8+ perforin expression of 88.4% and granzyme B expression of 91.92%, indicating marked activation of CD8+ cytotoxic T lymphocytes and pronounced reduction of NK cells. In addition, immunological assessment at admission revealed elevated IL-10 (160.94 pg/mL) and IFN-γ (24.65 pg/mL) levels, along with an IL-6 of 104.06 pg/mL and IL-8 of 103.05 pg/mL. Hemophagocytosis was not observed on bone marrow examination. However, based on the presence of fever, cytopenia, markedly elevated ferritin, elevated sIL-2R, splenomegaly, and hypofibrinogenemia, the patient fulfilled the HLH-2004 diagnostic criteria and was diagnosed with secondary HLH (12). The patient was treated with ABCD complex at escalating doses: 50 mg/day (approximately 0.7 mg/kg/day) for 2 days, 100 mg/day(1.3 mg/kg/day) for 1 day, and 150 mg/day(2.0 mg/kg/day) for 12 days. The cumulative ABCD dose during hospitalization was 2,000 mg (26.7 mg/kg). During treatment, the patient’s hemoglobin dropped from 101 g/L on admission to 56 g/L on day 7, with no definite bleeding source identified. He received packed red blood cell transfusions (2 units each) on day 8 and day 12, but hemoglobin improvement was not significant. He also continued to experience intermittent fever with a maximum temperature of 38.8 °C, suggesting that the HLH-related inflammatory response was not fully controlled. In addition to anti-parasitic therapy, methylprednisolone sodium succinate (MP, 40 mg/day) was administered for 7 days to control the inflammatory response. After treatment, body temperature gradually normalized, and the patient was discharged in stable condition. Follow-up laboratory tests at discharge showed: white blood cell count 2.4×10^9^/L, hemoglobin 83 g/L, platelet count 218×10^9^/L; C-reactive protein 1.23 mg/L; procalcitonin (whole blood) <0.05 ng/mL; ferritin 764.1 ng/mL; alanine aminotransferase 88.8 IU/L, aspartate aminotransferase 42.3 IU/L, albumin 24.9 g/L; serum creatinine 80.9μmol/L; fibrinogen 1.68 g/L; and D-dimer 5,511.0 ng/mL. **Figure 2** summarizes the clinical course of Case 2, including changes in body temperature and platelet count. The key clinical characteristics, treatment, and outcomes of both patients are also summarized in **Table 1** for easy reference.

## Discussion

Herein, we present two cases of VL complicating immunosuppressive therapy in patients with MN. The clinical course of these patients was consistent with the characteristic features of VL in immunocompromised hosts as described in the literature, yet also exhibited remarkable severity and complexity. Both patients shared similar backgrounds: they were middle-aged to elderly males with a history of MN, had received combination therapy with glucocorticoids and immunosuppressive agents, and presented with fever and pancytopenia as the predominant manifestations. However, despite some shared clinical features, the patients experienced distinct clinical outcomes. Case 1 initially improved after treatment but experienced early disease relapse and ultimately succumbed to multi-organ failure. In contrast, despite developing HLH, Case 2 achieved complete recovery following timely recognition and appropriate management. These contrasting outcomes highlight the clinical complexity of VL in immunosuppressed patients. Recent reviews have highlighted that VL in immunosuppressed patients presents with more severe manifestations, a more complex clinical course, and a more limited response to conventional therapies, underscoring the importance of individualized management strategies in this vulnerable population (3).

The primary factor contributing to the divergent outcomes of the two patients may be related to their immunosuppressive status before and after diagnosis. Previous literature has reported the occurrence of VL characterized by fever and pancytopenia in patients receiving immunosuppressive therapy for other conditions, including Crohn’s disease (21), multiple sclerosis (22), rheumatoid arthritis (23), and solid organ transplant recipients (24, 25). A common feature of these reports and the present cases is that immunosuppression due to the underlying disease is a key risk factor for the development and progression of VL. However, the unique feature of our cases is that both patients had MN and were receiving long-term immunosuppressive therapy for this condition. Such cases of VL complicating MN are rarely reported in the existing literature. Although all immunosuppressive agents were discontinued after admission in Case 1, his long-term use of leflunomide (20 mg/day) may have exerted a sustained immunosuppressive effect. Leflunomide, a pyrimidine synthesis inhibitor, selectively inhibits the proliferation of activated T lymphocytes by blocking the *de novo* pyrimidine synthesis pathway via inhibition of dihydroorotate dehydrogenase (26). The active metabolite, teriflunomide, has a long half-life of 15–18 days and undergoes significant enterohepatic circulation, leading to repeated drug reabsorption and further prolonging the duration of immunosuppression (26, 27). Moreover, the protein binding rate of teriflunomide exceeds 99%, meaning that even after drug discontinuation, it can persist in plasma for several weeks (28). In Case 1, the patient’s CD4+ T-cell count at the time of VL diagnosis was 247 cells/μL, indicating moderate-to-severe immunosuppression. In contrast, Case 2 had discontinued all immunosuppressive agents (tacrolimus and prednisone) 20 days before disease onset, and by the time of admission, the drugs had been largely metabolized and cleared, allowing partial recovery of the immune system, which created a more favorable immunological environment for anti-parasitic therapy. Clearance of *Leishmania* parasites critically depends on T cell-mediated Th1-type immune responses, particularly the activation of macrophages by interferon-gamma (IFN-γ) produced by CD4+ T cells to kill intracellular parasites (29). When Th1 immune function is impaired by immunosuppression, parasite clearance is compromised, leading to a significantly increased risk of treatment failure or relapse. Previous studies have reported that the one-year relapse rate of VL in HIV-coinfected patients ranges from 30% to 60%, and the initial treatment failure rate in solid organ transplant recipients is approximately 16%, with relapse rates significantly higher than those in immunocompetent individuals (cure rate of 93%–97% in the latter). These observations are consistent with the contrasting outcomes in our two cases, suggesting that differences in immunosuppressive drug metabolism and withdrawal timing may have contributed to the divergent prognoses.

In addition, both patients exhibited hyperinflammatory manifestations during their clinical course, including markedly elevated ferritin levels, suggesting the need for vigilance against severe complications such as HLH (14). VL is a well-recognized trigger of secondary HLH (14, 15). The underlying pathogenesis involves persistent antigenic stimulation by *Leishmania*, leading to excessive activation of T cells, NK cells, and macrophages, which in turn triggers a “cytokine storm” characterized by massive release of pro-inflammatory cytokines such as TNF-α, IL-6, and IL-10 (14, 15, 30). This overwhelming inflammatory response not only causes tissue damage but also suppresses bone marrow hematopoiesis, thereby exacerbating cytopenia (14, 15). Timely recognition of HLH and individualized management—including anti-parasitic therapy alone or with adjunctive immunomodulatory therapy—may improve prognosis (14). In Case 2, the patient met the HLH-2004 diagnostic criteria concurrently with the diagnosis of VL. Immunological assessment showed marked CD8+ T cell activation (perforin 88.4%, granzyme B 91.92%) and a markedly decreased NK cell count (1.01%). This immune profile, characterized by marked CD8+ T cell activation and NK cell reduction, has been described in secondary HLH (9, 10). In addition, elevated IL-10 (160.94 pg/mL) and IFN-γ (24.65 pg/mL) levels at admission, along with an IL-6 of 104.06 pg/mL, were consistent with the hyperinflammatory state of HLH. In the pathophysiology of HLH, CD8+ T cells exhibit persistent activation. Macrophages are activated by secreted IFN-γ, which in turn stimulates the release of IL-12 and IL-18, further promoting CD8+ T cell activation and forming a self-amplifying positive feedback inflammatory loop (31). Persistent antigen stimulation and relative insufficiency of cytotoxic function collectively lead to failure of activation-induced cell death (AICD), trapping CD8+ T cells in a state of sustained activation and preventing physiological termination of the immune response (10). In the present case, the high expression of perforin (88.4%) and granzyme B (91.92%) in CD8+ T cells is consistent with their activated state. Under these circumstances, even after anti-parasitic therapy is initiated, the already activated CD8+ T cells may not be efficiently eliminated through the defective AICD pathway; if left uncontrolled, this may lead to persistent inflammatory tissue damage. Therefore, glucocorticoids were added to the anti-parasitic regimen—in this case, methylprednisolone sodium succinate — to directly suppress the overactivated T cells and cytokine storm. The subsequent normalization of body temperature and clinical recovery supported the potential benefit of this intervention. This case suggests the importance of individualized treatment in VL-associated HLH. While some patients achieve remission with anti-parasitic therapy alone, critically ill patients—such as those with multi-organ dysfunction—may require adjunctive immunomodulatory therapy (14).

The significant difference in baseline renal function between the two patients may have been at least as influential as immunosuppressive status in determining the divergent outcomes. Case 1 had pre-existing chronic renal insufficiency on admission (serum creatinine 633.8 μmol/L), whereas Case 2 had essentially normal renal function (creatinine 90.4 μmol/L). During treatment, Case 1 exhibited marked fluctuations in serum creatinine, eventually progressing to end-stage renal disease requiring dialysis, and ultimately died of uremia combined with circulatory failure, suggesting that renal function status may be a key determinant of prognosis in VL. Renal insufficiency and VL can form a vicious cycle. On the one hand, VL itself can cause renal injuries such as glomerulonephritis and acute tubular necrosis (20), through mechanisms involving circulating immune complex deposition, cryoglobulinemia, and complement system activation (32). In immunocompromised patients, direct invasion of the renal parenchyma by the parasite also represents an important pathway of injury. On the other hand, prerenal factors (e.g., dehydration), sepsis, and the nephrotoxicity of anti-leishmanial drugs can all induce or exacerbate acute kidney injury (20),while renal insufficiency in turn impairs drug excretion and exacerbates toxin accumulation, thereby compromising the efficacy of anti-parasitic therapy and completing a vicious cycle. In this context, the nephrotoxicity of anti-leishmanial drugs themselves becomes a critical concern, particularly in patients with pre-existing renal impairment. The nephrotoxicity of amphotericin B primarily results from its preferential binding to cholesterol in the renal tubular epithelial cell membrane and subsequent induction of apoptosis (33). Consequently, patients with renal insufficiency are more susceptible to drug-induced kidney injury due to diminished renal reserve. Furthermore, antimonial agents commonly used to treat VL (e.g., sodium stibogluconate and meglumine antimoniate) are also associated with serious adverse effects, including nephrotoxicity, cardiotoxicity, hepatotoxicity, and pancreatitis (34). The above mechanisms suggest that anti-leishmanial agents should be carefully selected and individualized treatment regimens should be developed in patients with renal insufficiency: first, priority should be given to liposomal formulations of amphotericin B, which have relatively lower nephrotoxicity, over conventional amphotericin B; second, close monitoring of renal function and electrolytes should be performed during treatment; and third, renal replacement therapy should be considered when necessary to create favorable conditions for anti-infective treatment.

The early relapse observed in Case 1 within five days of discharge raises important questions regarding treatment adequacy and the possibility of persistent parasitic burden. During the first admission, the patient received a cumulative ABCD dose of 1,400 mg (23.1 mg/kg), which reached the lower threshold of the 20–60 mg/kg range recommended for immunocompromised patients according to the Chinese Expert Consensus on the Diagnosis and Treatment of Leishmaniasis (2017) (35). Although this dose exceeds the WHO-recommended cumulative dose of up to 15 mg/kg for immunocompetent patients, it may still be insufficient for a patient with prolonged immunosuppression. This possibility is supported by the findings at readmission: bone marrow smear showed no visible Leishman-Donovan bodies, yet PCR remained positive for *Leishmania* DNA. This discordance between microscopy and PCR suggests that parasite burden had been substantially reduced but not completely eradicated, allowing residual parasites to reactivate shortly after treatment cessation. Consistent with this, Melkamu et al. reported that among microscopically negative tissue smears, PCR was positive in 62.3% of cases, and the rate was even higher (82.1%) in bone marrow samples (36). These findings suggest that PCR may be more sensitive than microscopy for detecting residual *Leishmania* infection, and its use could be considered in monitoring patients at risk of relapse. This case also suggests that higher cumulative doses or extended treatment courses may be required to achieve parasitological cure in patients with prolonged immunosuppression.

This study has several limitations. First, this report includes only two cases, and the limited sample size precludes statistical inference. Second, parasitological cure was not confirmed by post-treatment bone marrow examination in either case, as discharge decisions were based on clinical cure criteria rather than parasitological confirmation. Third, detailed parasite drug susceptibility data for Case 1 were lacking, so the specific mechanism underlying treatment failure remains unclear. Fourth, immunological data—including NK cell count, perforin, and granzyme B expression—were available only for Case 2. In addition, while cytokine levels were measured, functional assays such as antigen-stimulated IFN-γ production and T-lymphocyte proliferation were not performed in either case, limiting the interpretation of immune function. Future studies with larger sample sizes, incorporating more comprehensive immunological and parasitological monitoring, are needed to optimize individualized multidisciplinary management strategies for such high-risk patients.

## Conclusion

These two cases suggest that patients with MN receiving long-term immunosuppressive therapy may be at increased risk for VL and may face multiple treatment challenges, including pre-existing renal insufficiency, prolonged immunosuppression, and HLH. Our observations highlight the importance of comprehensive assessment of these factors and the need for individualized strategies—including careful adjustment of immunosuppressants, preference for less nephrotoxic drugs, and timely recognition and management of HLH. These experiences point to the potential benefit of multidisciplinary collaboration and continuous monitoring in the care of such complex patients.

## Data Availability

The original contributions presented in the study are included in the article. Further inquiries can be directed to the corresponding authors.
